# Interstitial Lung Disease in Common Variable Immunodeficiency

**DOI:** 10.3389/fimmu.2021.605945

**Published:** 2021-03-11

**Authors:** Joao Pedro Lopes, Hsi-en Ho, Charlotte Cunningham-Rundles

**Affiliations:** ^1^Division of Allergy, Immunology and Rheumatology, Department of Pediatrics, UH University Hospitals Rainbow Babies and Children, Cleveland, OH, United States; ^2^Division of Clinical Immunology, Department of Medicine, Icahn School of Medicine at Mount Sinai, New York, NY, United States

**Keywords:** common variable immune deficiency (CVID), interstitial lung disease (ILD), autoimmunity, lung transplant, cytopenia, malignancy, lymphoma

## Abstract

Interstitial lung disease (ILD) is a common complication in patients with common variable immunodeficiency (CVID) and often associated with other features, such as bronchiectasis and autoimmunity. As the ILD term encompasses different acute and chronic pulmonary conditions, the diagnosis is commonly made based on imaging features; histopathology is less frequently available. From a cohort of 637 patients with CVID followed at our center over 4 decades, we reviewed the data for 46 subjects (30 females, 16 males) who had lung biopsies with proven ILD. They had a median age at CVID diagnosis of 26 years old, with a median IgG level at diagnosis of 285.0 mg/dL with average isotype switched memory B cells of 0.5%. Lung biopsy pathology revealed granulomas in 25 patients (54.4%), lymphoid interstitial pneumonia in 13 patients (28.3%), lymphoid hyperplasia not otherwise specified in 7 patients (15.2%), cryptogenic organizing pneumonia in 7 patients (15.2%), follicular bronchitis in 4 patients (8.7%), and predominance of pulmonary fibrosis in 4 patients (8.7%). Autoimmune manifestations were common and were present in 28 (60.9%) patients. Nine patients (19.6%) died, with a median age at death of 49-years-old. Lung transplant was done in 3 of these patients (6.5%) who are no longer alive. These analyses reveal the high burden of this complication, with almost one-fifth of the group deceased in this period. Further understanding of the causes of the development and progression of ILD in CVID patients is required to define the best management for this patient population.

## Introduction

Common variable immunodeficiency (CVID) is the most prevalent form of clinically-recognized primary immunodeficiency, characterized by low serum IgG levels, usually a low IgA, and often a low IgM, reduced or absent antibody responses to disease or immunizations. This defect leads to recurrent infections, with particular emphasis on the sinorespiratory tract (1–6). CVID is also commonly associated with inflammatory complications, leading to chronic lung disease, generalized lymphoid hypertrophy, splenomegaly, gastrointestinal disease, and cytopenias, amongst other inflammatory manifestations (7–11). Interstitial lung disease (ILD) is a term that encompasses a group of different acute and chronic pulmonary conditions with common clinical and physiological characteristics. This condition is a common complication in patients with CVID. The diagnosis of ILD is commonly made based on clinical presentation and includes characteristic imaging features. For those in whom a biopsy was performed, histology provides further confirmation of the diagnosis, along with the individual pathologic features (12–14).

Chronic lung disease, including ILD, is often associated with other inflammatory features, such as lymphoid hyperplasia and autoimmunity. When present, the lung damage is associated with shortened survival; this has been noted as the leading cause of death in some CVID cohorts (5, 7, 9, 15–22). Previous publications have addressed the frequency of clinically diagnosed ILD in CVID, noted in the 10–20% range (15–18, 23). Bates et al. on a cohort of 69 CVID patients showed reduced survival for ILD vs. non-ILD patients (17). In this study, the ILD diagnosis was associated with a propensity for T cell lymphopenia, splenomegaly, and restrictive pulmonary physiology (17). Relative lymphopenia was also noted in data on a cohort from the USIDNET (United States Immunodeficiency Network) Registry; here, Kellner et al. analyzed data from 1,518 CVID patients, of whom 138 patients (9.1%) had an ILD diagnosis. These patients had lower CD3, CD4, and CD8 T cell counts than patients without ILD, suggesting an increased risk of complications related to these abnormalities (18).

While the pathogenesis of ILD in a significant number of CVID patients remains unclear, genetic defects, and T cell and B cell dysregulation have been associated with progression. As suggested by Weinberger et al. based on comparing patients with X-linked agammaglobulinemia (XLA) to CVID patients in the USIDNET Registry, lack of antibody alone would not appear to be the leading cause of ILD, as a higher frequency of ILD, as well as of respiratory infections and asthma, was described in CVID when compared to patients with XLA (24).

In the absence of a consensus in terms of the best therapeutic approach for CVID patients with ILD (25), several therapeutic options have been discussed with an attempt to control, even if not to reverse, the progression of ILD in CVID patients (20). These include rituximab, corticosteroids, and a number of other immunosuppressive agents (26–28).

Here we review data on a group of patients in our New York CVID cohort who had biopsy-proven ILD, examining their clinical, laboratory, radiologic, histopathological, and functional data. We also aimed to review the existing data on the spectrum of ILD in CVID, to put our findings in perspective of the joint efforts by other groups to better understand this presentation's pathophysiology and potential avenues for better prevention and treatment in the near future.

## Materials and Methods

### Patients

A cohort of 637 subjects with CVID (337 females and 285 males) were seen at Mount Sinai Medical Center from 1986 through the present. The first part of the cohort was previously seen at Memorial Sloan-Kettering Cancer Center (1974–1986); subsequently, these subjects were seen at Mount Sinai Medical Center. The diagnosis of CVID was made by standard criteria, including reduced serum IgG, IgA, and/or IgM, by at least 2 SDs below the mean for age, with poor or absent antibody production to both protein and carbohydrate vaccines and exclusion of other causes of hypogammaglobulinemia. Subjects under age 4 years without continued follow-up and subjects with lymphoid cancer diagnosed within 2 years after the diagnosis of CVID were excluded. For the 46 patients with biopsy-proven ILD, medical, radiologic immunologic, and pathology data were reviewed for this report.

### Immunologic Parameters

Enumeration of T and B cells, CD4, and CD8 T cells, and IgM^−^IgD^−^CD27^+^ isotype switched memory B cells as a proportion of total B cells were determined.

### Data

Data was abstracted in Microsoft Excel and analyzed in IBM SPSS Statistics. All studies were undertaken with the consent of the Mount Sinai Medical Center Institutional Review Board.

## Results

### Demographics and Immune Phenotypes

Forty-six patients with biopsy-confirmed ILD and for whom pathology reports were available, included 30 females and 16 males. Two patients were African American; the rest were Caucasian. The age at CVID diagnosis was 26 years (range 1.0–66.0 years old), with lung symptoms appearing, as noted in the chart, at a median age of 29 years (range 1.0–59.0 years old). Immunoglobulin replacement was started later, at a median age of 32.5 years (Table 1). Baseline immunoglobulins, IgG, IgA, and IgM, are noted in Table 2, with the values presented in the Table for this group of 46 patients with CVID and ILD, similar to 500 other CVID subjects in this cohort with no known ILD (IgG = 246+/−221 mg/dL; IgA = 7.0+/−30.4 mg/dL, and IgM=20+/−166.4 mg/dL) (Mann-Whitney test). Absolute CD3, CD4, CD8 T cells, and CD19 B lymphocyte numbers were overall within normal limits but with wide variation. The percent of isotype switched memory B cells were low, as characteristic of CVID subjects (Table 2).

**Table 1:** Demographic information of CVID patients with ILD included in the study.

**Age (46 subjects, 30 females, 16 males)**	
CVID diagnosis (years-old; median, SD)	26.0 (17.8)
Onset lung symptoms (years-old, median, SD)	29.8 (16.5)
Ig replacement (years-old, median, SD)	32.5 (17.5)

**Table 2:** Immunologic laboratory data of CVID patients with ILD.

**Immunoglobulins**	**(Median +/– SD)**
Baseline IgG (normal 700–1,600 mg/dL)	**285.0** mg/dL (232.0)
Baseline IgA (normal 90–386 mg/dL)	**6.5** mg/dL (11.2)
Baseline IgM (normal 20–172 mg/dL)	25.5 mg/dL (59.0)
**Lymphocytes** **(average count, SD)**	**(median +/– SD)**
ABS CD3 T cells (normal 575–2,237/μL)	898.0 (714.8) range 278–4232
ABS CD4 T cells (normal 325–1,472/μL)	555.0 (476.1) range 203–2828
ABS CD8 T cells (normal 109–897/μL)	366.0 (287.6) range 62–1404
ABS B cells (normal 12–645/mm3)	83.4 (128.7) range 0–512
Isotype switched CD27+B cells (% of B cells] (normal 10–22.2%)	**0.5%** (1.4%) range 0–5.8%

*Values bold when outside the normal reference range*.

### Pathology

**Figure 1:**
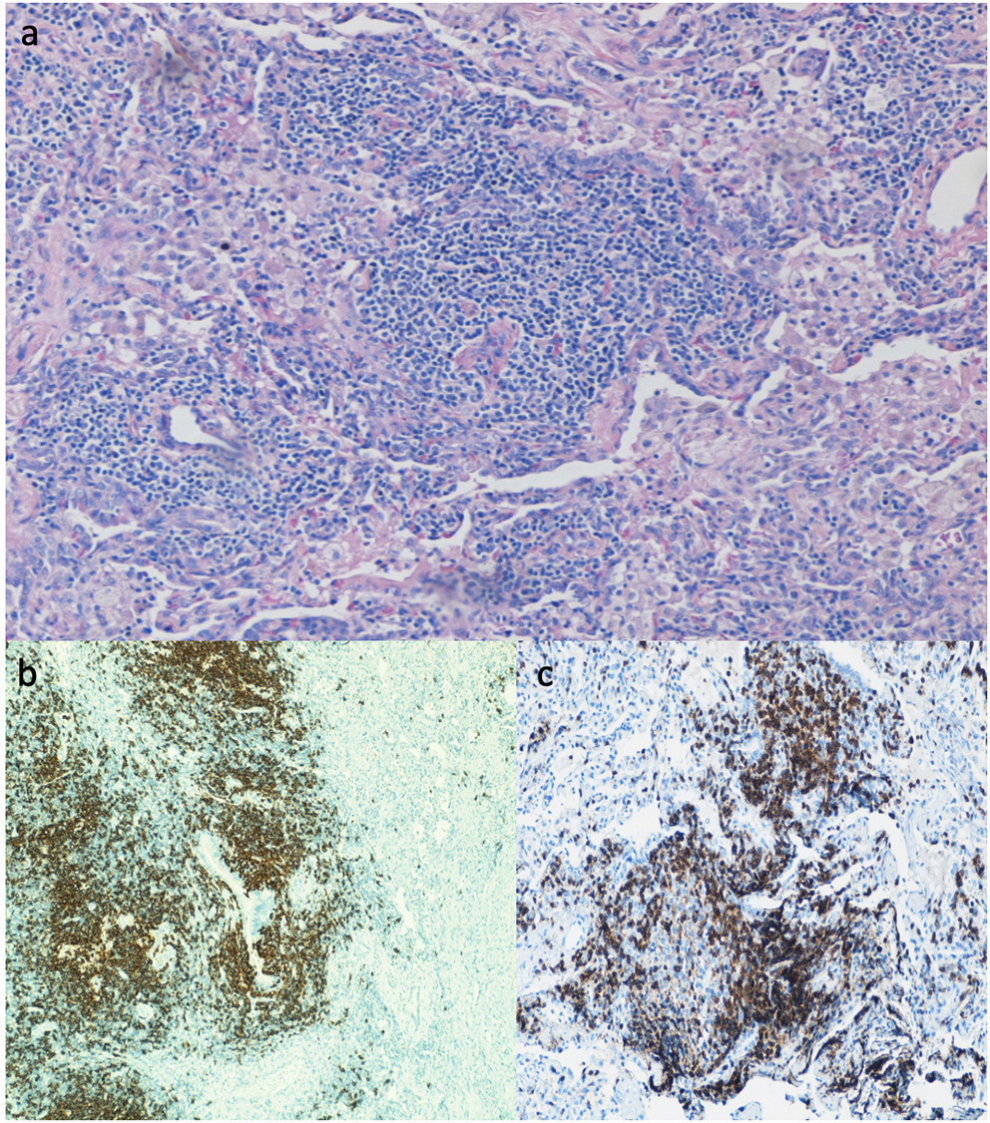
Lung biopsy of CVID patient with ILD, showing the presence of a granuloma lesion, with presence of lymphocytes on H&E staining **(a)**, immunohistochemistry for CD20 **(b)**, and for CD3 cells **(c)**. Magnification of images is 40x.

Lung biopsy pathology revealed granulomatous infiltrates in 25 of the 46 patients (54.3%), lymphoid interstitial pneumonia in 13 (28.3%), lymphoid hyperplasia not otherwise specified in 7 (15.2%), cryptogenic organizing pneumonia in 7 (15.2%), follicular bronchitis in 4 (8.7%), and predominance of pulmonary fibrosis in 4 patients (8.7%). Combinations of these pathologic findings were found in several subjects (Table 3). Figure 1 contains the lung biopsy of one of the ILD/CVID patients in this group, demonstrating the presence of a granulomatous lesion with lymphocytic infiltration. Bronchiectasis was concomitantly described in 8 (17.4%) patients.

**Table 3:** Lung pathology encountered in the CVID patients with ILD.

	**Number**	**Percent**
Granulomas	17	37
LIP	7	15.2
COP	4	8.7
Fibrosis	3	6.5
LIP, granulomas	3	6.5
Lymphoid hyperplasia	3	6.5
LIP, follicular bronchiolitis	2	4.3
COP, fibrosis	1	2.2
COP, granulomas	1	2.2
Follicular bronchiolitis, lymphoid hyperplasia, granulomas	1	2.2
LIP, COP, granulomas	1	2.2
Lymphoid hyperplasia	1	2.2
Lymphoid hyperplasia, granulomas	1	2.2
Lymphoid hyperplasia, granulomas, follicular bronchiolitis	1	2.2
Total	46	100

*COP, Cryptogenic organizing pneumonia; LIP, lymphocytic interstitial pneumonia*.

### Lung Functions

Results available for 28 patients (60.9%) revealed the group to have an average FEV1/FVC ratio of 0.85 (standard deviation of 0.11; normal ratio above 0.75), with an average FEV1 of 0.71 (standard deviation of 0.17; normal FEV1 above 0.80) and FVC of 0.72 (standard deviation of 0.18; normal FVC above 0.80). The average TLC was 0.78 (standard deviation of 0.14; normal TLC above 0.80) and the average DLCO was 60.8% of predicted (standard deviation 22.3%, range 16.0 to 109.0%; normal DLCO above 75.0% of predicted).

### Radiologic Studies

**Figure 2:**
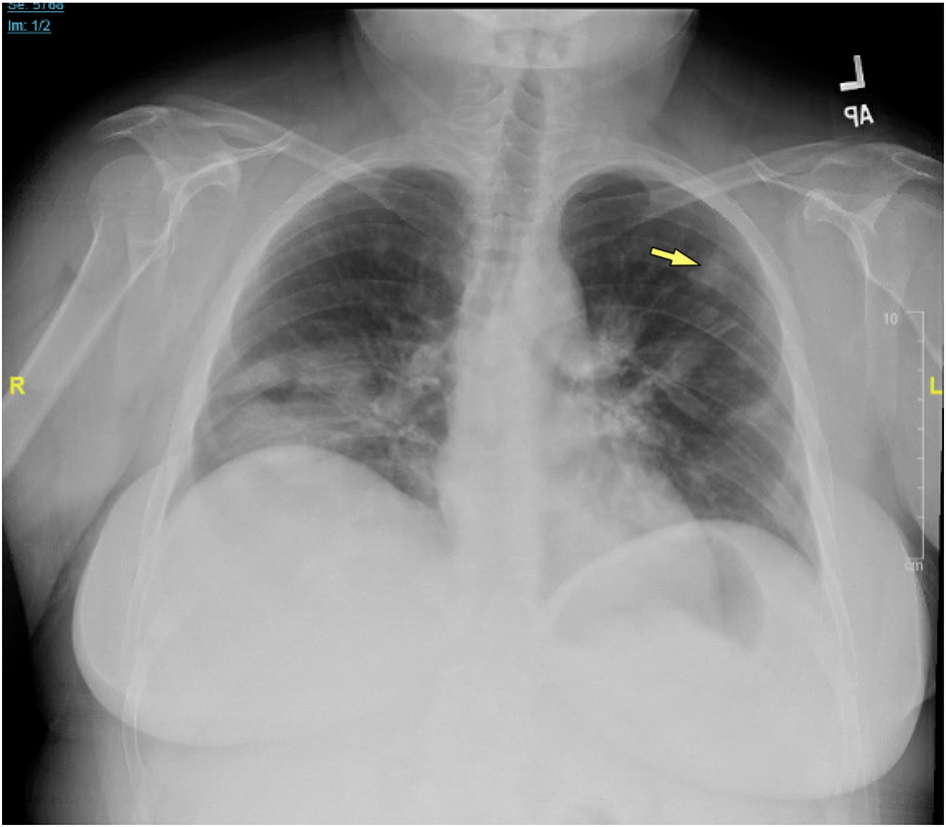
Chest x-ray of CVID/ILD patient demonstrating presence of 1.6 cm lesion in the left upper lobe (yellow arrow), as well as patchy densities in the mid to lower lung fields.

**Figure 3:**
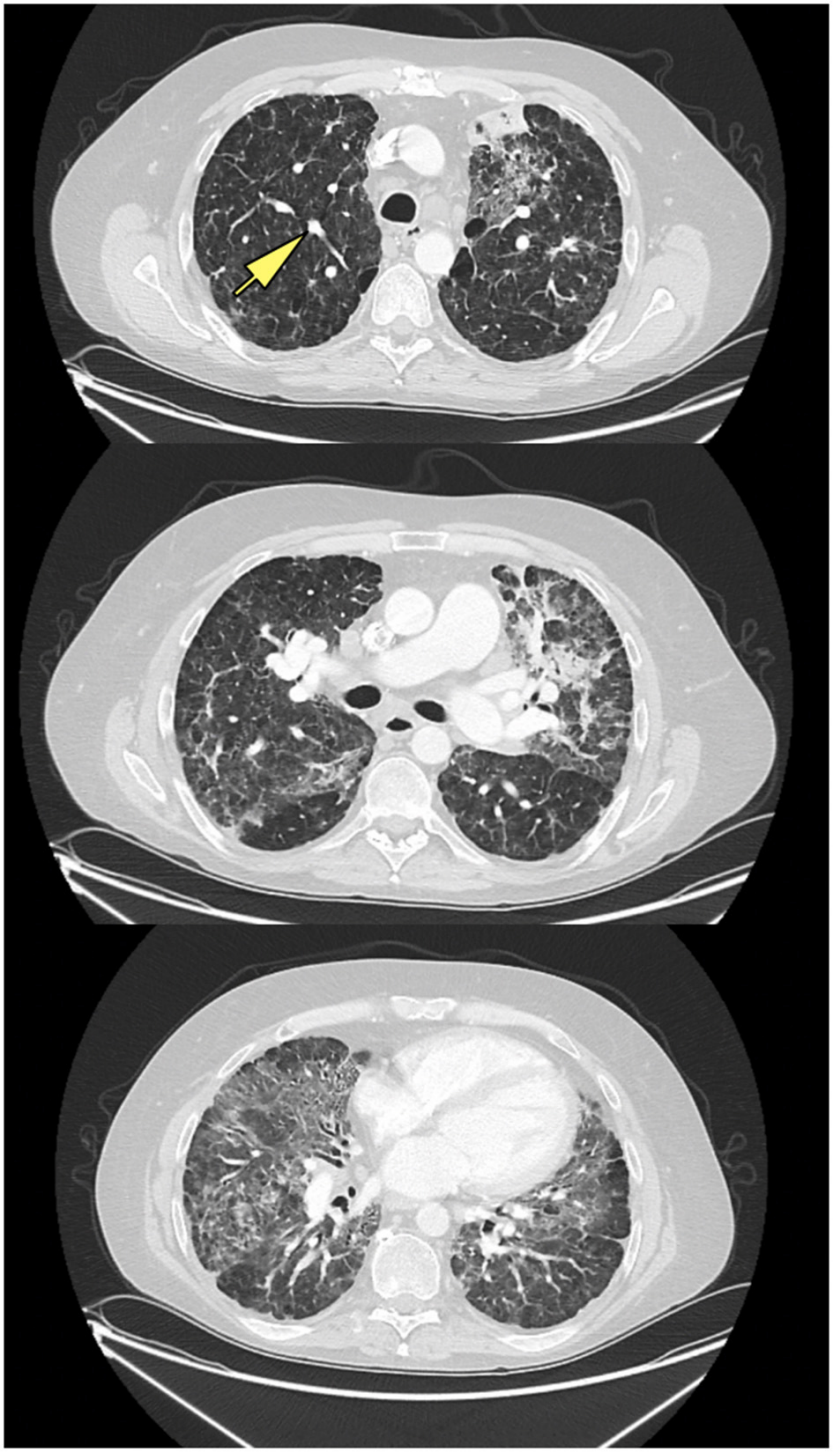
CT scan of ILD/CVID patient, with upper, middle, and lower lung zones, demonstrating mid to lower lung zone predominant ground glass opacities, within a bronchovascular distribution, with associated volume loss, with left upper lobe consolidation in association with air bronchograms, likely pneumonia, new solid nodular opacity (12 × 7 mm) (yellow arrow) in the right upper lobe, as well as bilateral hilar lymphadenopathy.

Chest x-rays were available for 15 patients (32.6%), describing the presence of nodular opacities in 12 patients (80.0%) reticular infiltrates in 6 patients (40.0%), and fibrosis in 2 patients (13.3%). Computerized tomography scans of the chest were available for 32 patients (69.6%). Findings were notable for the presence of nodules in 30 patients (93.8%), mediastinal lymphadenopathy in 21 patients (65.6%), ground-glass appearance in 12 patients (37.5%), diffuse consolidation in 4 patients (12.5%), granulomas in 2 patients (6.3%), and fibrosis in 1 patient (3.1%). Examples of radiologic findings are shown in Figure 2 (chest x-ray) and Figure 3 (chest CT scan).

### Clinical Features

Autoimmune manifestations other than in the lung were present in 28 (60.9%) patients, with 12 of those patients (26.1%) having more than one autoimmune manifestation. Cytopenias were a common manifestation: immune thrombocytopenic purpura (ITP) in 18 patients (39.1%), autoimmune hemolytic anemia (AIHA) in 9 patients (19.6%), autoimmune neutropenia in 6 patients (13.0%), pancytopenia in 3 patients (6.5%), and red blood cell aplasia in 2 patients (4.3%). Many had more than one of these conditions, most commonly, AIHA and ITP. Other conditions included uveitis, severe aphthous ulcers, primary biliary cholangitis, and rheumatoid arthritis in one patient each. Twenty nine patients (63.0%) were observed to have lymphadenopathy, and the same number to have splenomegaly. Splenectomy had been done in 10 of these patients (21.7%). Nodular regenerative hyperplasia of the liver was noted in 7 patients (15.2%). Five of these patients (10.9%) developed a malignancy, with 4 (8.7%) developing a lymphoma. One other patient had ovarian cancer.

Unusual infections were identified in several of these patients: 5 patients (10.9%) had herpes zoster (caused by the varicella zoster virus), 1 patient (2.2%) had atypical mycobacteria lung infection, 1 patient (2.2%) had measles encephalitis, 1 patient (2.2%) had metapneumovirus infection, and 1 patient (2.2%) had *Pseudomonas* otitis complicated by *Pseudomonas* bacteremia.

### Genetics

A gene mutation associated with or contributing to the patient's CVID phenotype was identified in 10 of the 31 subjects available for testing by whole-exome sequencing (32.3%); 3 patients (9.7%) had a *CTLA4* mutation, 2 patients (6.5%) had an *NFKB1* mutation, 2 patients (6.5%) had either one or two TACI (*TNFRSF13B*) mutations, and 1 each had a *STAT3* mutation, *KMT2D* mutation, or a *PIK3CD* mutation (3.2%).

### Treatment and Outcomes

Treatment modalities used in these subjects are outlined in Table 4. Seven patients (15.2%) required chronic oxygen supplementation, and 5 patients (10.9%) were diagnosed with pulmonary hypertension. Lung transplant had been done in 3 of the patients described here (6.5%); none are currently surviving (Table 5). Overall, 9 of these patients (19.6%) have died, with a median age of death of 49.0 years-old (range 27.0–70.0 years-old, standard deviation of 15.1 years).

**Table 4:** Treatment modalities used in the CVID patients with ILD in the study.

Corticosteroids	23 patients (50.0%)
Rituximab	16 patients (34.8%)
Mycophenolate mofetil	5 patients (10.9%)
Azathioprine	4 patients (8.7%)
Mercaptopurine	2 patients (4.3%)
Hydroxychloroquine	2 patients (4.3%)
Abatacept	1 patient (2.2%)
Sirolimus	1 patient (2.2%)
Cyclosporine	1 patient (2.2%)

**Table 5:** Lung transplant characteristics in this cohort.

	**Patient 1**	**Patient 2**	**Patient 3**
Year born	1959	1963	1949
Lung pathology	Chronic obstructive pulmonary disease	Pulmonary fibrosis predominates with granuloma	ILD (granuloma and lymphoid infiltrate), bronchiectasis
CVID-associated comorbidities	Enteropathy	Liver disease	None
Transplant procedure	Lung and heart	Lung	Lung
Year of transplant procedure (age)	1983 (age 34)	1997 (age 34)	2018 (age 70)
Outcome	Died of chronic rejection after 5 years	Operative complications, died of hyperacute rejection within a week	Died of acute rejection after 8 months; CMV infection

## Discussion

We describe 46 patients with biopsy-characterized ILD in our cohort of 637 CVID patients, 7.2% of the cohort. As recently published, based on both radiologic studies and pathology, the overall frequency of ILD in our CVID cohort is 10.4% (15), similar to other reports in which the incidence ranges from 10 to 20% in CVID (16, 18, 23, 29). In this report, we focus on the subjects for whom a biopsy had been done to provide further pathology.

A study by Patel et al. on data from the Oxford Primary Immune Deficiencies Database evaluated lung biopsies from 16 CVID patients, recognizing the presence of lymphocytic infiltrations as the most common pattern. In the Oxford report, 5 of these patients were also evaluated with immuno-markers, showing T cell infiltrates in 4 patients and B cell infiltrates in one other individual (30). In contrast, analysis of the lung biopsy results in our group demonstrated granulomatous infiltration in more than half of our patients. The commonly used term of granulomatous-lymphocytic interstitial lung disease (GLILD) can be applied to these subjects. Lymphoid infiltrations were the second most prevalent condition, found in 20 patients.

Almost all patients had a description of numerous lung nodules from the radiologic perspective, and nearly two-thirds had mediastinal lymphadenopathy. More than one third had areas with ground-glass appearance. Only 2 patients had “granulomas” suggested on their CT report. As more than half of the patients had granulomas present in biopsies, it is clear that, from the CT perspective, this form of pathology would not be clarified by radiologic observations. As previously suggested (31), it is important not only to recognize specific CT patterns of ILD but also early lung abnormalities at a subclinical level. Not surprisingly, patients had impaired lung functions with reduced FEV1, FVC, TLC, and DLCO, all in line with a restrictive disease pattern previously described in similar cohorts (17).

In previous studies, B cell dysregulation has been associated with progression of ILD. Maglione et al. analyzed CVID patients with ILD treated with rituximab, noting that recurrence of lung disease was associated with an increase of B cell-activating factor (BAFF) in the peripheral blood; this could potentially lead to the B cell hyperplasia in the lung, with the development of germinal centers as one driver of lung damage in these patients (32). In the same paper, progression of ILD, as well as ILD recurrence post-rituximab, were also seen to be associated with increasingly elevated serum IgM, potentially a reflection of the increasing hyperplasia of local pulmonary B cell follicles (32). However, for the 46 biopsied subjects examined here, serum IgM was not different from 500 other subjects in this cohort without confirmed ILD.

In previous ILD studies, patients have commonly been noted to have many additional inflammatory complications (33). In our group, splenomegaly and lymphadenopathy were present in nearly two-thirds of the patients, and more than half had had cytopenias (mostly ITP, but also AIHA or neutropenia). In one cohort of 105 adult CVID patients, more patients had splenomegaly (74.0%) and lymphadenopathy (63%) than non-ILD patients (16). Maglione et al. also reviewed CT imaging from CVID patients with pulmonary disease; here, while the presence of bronchiectasis was associated with a higher number of infections, imaging patterns of ILD were more frequently associated with autoimmunity and lymphoproliferation (21). The high frequency of splenomegaly and the history of cytopenias were also highlighted as potential predictors of granulomatous lymphocytic interstitial lung disease (GLILD) by Hartono et al. in a 2017 study (33). In our cohort, splenectomy had been performed in more than one-fifth of these patients for one or more of these cytopenias and/or hypersplenism. Noteworthy as well is that nodular regenerative hyperplasia of the liver, another inflammatory condition of unclear etiology, was documented in 7 of these patients (15.2%).

CVID patients are also known to have increased malignancy rates, particularly lymphoma, described with a rate of 1.6–8.2% of CVID patients, depending on the cohort (9, 19, 34). In this group, the rate was 8.7%, a remarkable reminder of the higher risk for lymphoma in this particular patient group and the importance of appropriate surveillance.

As for treatment, rituximab, a monoclonal antibody targeting CD20, with the goal of B cell depletion, has been successfully used in this patient population, as monotherapy or in combination with other immune suppressants (26, 32, 35, 36). Chase et al. examined combination therapy with rituximab and azathioprine in 7 patients, noting improvement in both pulmonary function and CT abnormalities, without significant treatment side effects (27). Corticosteroids have been one of the mainstays of ILD treatment (28), but as is well-documented, their long-term use is associated with side effects, some of them potentially severe (37). Other immunosuppressive medications, such as mercaptopurine, cyclosporine, hydroxychloroquine, mycophenolate mofetil, or abatacept, have been used, with variable success (38–40). Our group of patients had varying use of different immunosuppressive agents, with half of the group having documentation of corticosteroid use at some point and more than one third having received rituximab, but other agents such as mycophenolate mofetil or azathioprine were also used in this population. Additional data on the response of ILD to different agents will be necessary to, if not reaching a consensus, at least define the best available therapies to contain or reverse the progression of lung disease. More knowledge on the genetics and/or pathogenesis for each patient, may allow some ability to tailor these therapies more individually.

That almost one-fifth of the patients discussed here died with a median age of death of 49 years-old is a striking reminder of the shortened life expectation for CVID patients with ILD. This is also highlighted by the 7 patients requiring chronic oxygen and the 5 diagnosed with pulmonary hypertension requiring additional therapies. Lung transplantation has been done increasingly for several end-stage lung diseases, and post-transplant survival has improved in the last decades (41). The three patients in our group of 46 with biopsy-proven ILD who underwent lung transplant died. Parenthetically, out of the 637 CVID patients followed in our center, a total of 8 patients have now undergone lung transplant (three of these are part of the 46 patients in this cohort described in Table 5). Only one of these 8 patients submitted to lung transplant is now alive. It remains unclear for which CVID patients with end-stage respiratory disease this would be a viable option.

As our cohort spans almost 50 years of follow-up, only 31 of the 46 patients had a genetic evaluation, but in these, 10 had genes now identified as leading to or associated with this immune defect. In some cases (*CTLA4, STAT3*), these data may help in suggesting more targeted therapies for ILD (abatacept, or tocilizumab as an anti–IL-6 receptor mAb). The increasing use of genetic analysis has helped to better understand and define the CVID syndrome (42–45) and, hopefully, will lead to a better understanding of the pathogenesis and/or suggest new therapies.

## Data Availability Statement

The raw data supporting the conclusions of this article will be made available by the authors, without undue reservation.

## Ethics Statement

The studies involving human participants were reviewed and approved by Icahn School of Medicine at Mount Sinai Institutional Review Board. Written informed consent to participate in this study was provided by the participants' legal guardian/next of kin.

## Author Contributions

All authors participated in the data collection, data analysis, manuscript writing, and manuscript review of the research data here presented.

## Conflict of Interest

The authors declare that research was conducted in the absence of any commercial or financial relationships that could be construed as a potential conflict of interest.
